# Accelerated search for biomolecular network models to interpret high-throughput experimental data

**DOI:** 10.1186/1471-2105-8-258

**Published:** 2007-07-18

**Authors:** Suman Datta, Bahrad A Sokhansanj

**Affiliations:** 1 School of Biomedical Engineering, Science and Health Systems, Drexel University, 3141 Chestnut St., Philadelphia, PA 19104, USA

## Abstract

**Background:**

The functions of human cells are carried out by biomolecular networks, which include proteins, genes, and regulatory sites within DNA that encode and control protein expression. Models of biomolecular network structure and dynamics can be inferred from high-throughput measurements of gene and protein expression. We build on our previously developed fuzzy logic method for bridging quantitative and qualitative biological data to address the challenges of noisy, low resolution high-throughput measurements, i.e., from gene expression microarrays. We employ an evolutionary search algorithm to accelerate the search for hypothetical fuzzy biomolecular network models consistent with a biological data set. We also develop a method to estimate the probability of a potential network model fitting a set of data by chance. The resulting metric provides an estimate of both model quality and dataset quality, identifying data that are too noisy to identify meaningful correlations between the measured variables.

**Results:**

Optimal parameters for the evolutionary search were identified based on artificial data, and the algorithm showed scalable and consistent performance for as many as 150 variables. The method was tested on previously published human cell cycle gene expression microarray data sets. The evolutionary search method was found to converge to the results of exhaustive search. The randomized evolutionary search was able to converge on a set of similar best-fitting network models on different training data sets after 30 generations running 30 models per generation. Consistent results were found regardless of which of the published data sets were used to train or verify the quantitative predictions of the best-fitting models for cell cycle gene dynamics.

**Conclusion:**

Our results demonstrate the capability of scalable evolutionary search for fuzzy network models to address the problem of inferring models based on complex, noisy biomolecular data sets. This approach yields multiple alternative models that are consistent with the data, yielding a constrained set of hypotheses that can be used to optimally design subsequent experiments.

## Background

The functions of living cells are carried out by biomolecular networks: proteins that execute, assist, and provide structure for biochemical reactions, and genes and regulatory sites within DNA which encode protein structure and regulation. The connections and rules governing the dynamics of a biomolecular network can be identified by systematically studying how perturbations of particular genes and proteins in the network affect the levels and activities of others. High-throughput technologies can measure multiple genes and proteins simultaneously under a given set of experimental conditions, perturbations, or clinical context. This enables an inverse approach using data to infer models of the biomolecular network. Considerable attention has been paid to this inverse or "reverse engineering" problem in biology in recent years (see for example the reviews [1-4], among many others).

A crucial question in developing models of biomolecular networks, whether through a forward or inverse approach, is the choice of mathematical representation for network dynamics. The simplest approach is Boolean logic [5]; however, binary rules were recognized early on to lack the dynamic resolution and range necessary to model biological function [6]. At the other end of the spectrum of computational complexity are differential equations and other models based on chemical and physical interactions and dynamics [7]. Inverse methods for these kinds of physical models have been developed [8,9]. However, the increased resolution comes at the cost of requiring more accurate and comprehensive biochemical data to estimate model structure and dynamics. This is not feasible for the current state of high-throughput technologies in genomics and proteomics, such as microarrays and mass spectroscopy, which generate noisy, semi-quantitative data.

We have previously proposed and tested the utility of fuzzy logic as a semi-quantitative bridge between logical and physical models of biomolecular networks [10,11]. An additional advantage of fuzzy logic is the ability to use linguistic terms, allowing for a bridge to the current text and graphics-based models used by biological scientists. (The authors of [12] propose another method employing fuzzy logic for models of 3-gene sub-networks to analyze microarray data.) A related approach is qualitative simulation (reviewed in [13]). However, qualitative differential equations and similar implementations do not have a straight-forward, systematic connection to quantitative and qualitative data nor the convenient linguistic interpretability of fuzzy logic. Qualitative methods are also generally limited to what are essentially binary or ternary dynamics (i.e., increasing vs. decreasing) without any gradations between classes.

Our approach to fuzzy logic uses the fuzzy equivalent to the Boolean "OR" rather than "AND" [14]. This results in a more scalable framework of linear rules between biomolecular network components. Consequently, we can obtain multiple solutions for the inverse problem of model reconstruction based on data. This is not possible using more computationally complex continuous models (e.g., [9]). We thus avoid imposing the additional constraint of finding the sparsest possible network model, which may not correspond to the underlying biology, especially when in a context where multiple components of a network are highly active. Bayesian networks [15] and machine learning methods [16] have been proposed for solving the inverse problem. In principle, these would allow for evaluation of multiple models; in practice, they are employed to find the "optimal solution" (based on constraints such as experimental fit and sparsity). A method for developing multiple hypotheses using genetic algorithms and then ranking them was previously proposed [17]. However, the multimodal logic used in that implementation does not have the continuous granularity of fuzzy logic. Therefore, the method lacks resolution and is not compatible with quantitative data needed to model complex interactions.

There are two key factors that result in multiple solutions to the inverse problem of generating biomolecular network models based on data. (1) Networks being modeled typically have more components and interactions than data points being measured – and even when there are more data points, because of noisy data, there are still insufficient measurements for precise model estimation (i.e., the problem is undetermined). (2) Biomolecular networks are *abstractions*, and our identification of their components is necessarily limited. For example, mRNA microarrays measure the expression levels of genes. The co- or anti-expression of these genes suggests functional correlations, which can be termed gene regulatory networks (or "gene networks"). However, the expression of a gene relates to the rate of production of the protein it encodes. There is no information about the modification, activity, or interaction of these proteins with each other and other cellular components. Consequently, no "gene network model" will ever actually represent a "biological truth". Even combining all kinds of proteomic, genomic, metabolomic, etc., data and corresponding variables ot the model will inevitably leave out unidentified components, modifications, and interactions.

Thus, there is no "gold standard solution" for the biological reverse engineering problem. Nor does comparing models on the basis of their agreement with previous biological knowledge provide any "proof" of success. Both the mathematical abstractions of models and the linguistic abstractions used by a biologist interpreting results in qualitative language are necessarily incomplete. The correct goal for the biological reverse engineering method should not be the "actual" model, which is ill-defined at best, but rather multiple, alternative *plausible *models consistent with the data. The set of plausible models may then be used to perform further simulations, complement other models, interpret biological information, and pose hypotheses for experimental study.

We present an evolutionary algorithm method to accelerate the search for plausible biomolecular network fuzzy logic models. We focus on gene network models based on mRNA microarray data, but the approach has been designed to be flexibly employed on other kinds of data. Our objective is a method that can be employed to obtain rule-based models for gene expression dynamics in a 20–100 gene network identified using previous knowledge (the previous exhaustive search was limited to about 10 genes). Suggested parameters for the algorithm are estimated based on artificial data, and the method is demonstrated on multiple data sets for human cell cycle-regulated genes obtained through different synchronization techniques [18]. We also introduce a method for estimating of the probability that a rule governing a particular gene in a network model was obtained by chance, which provides an assessment of the redundancy of the multiple plausible solutions to the inverse problem for biomolecular networks.

## Results

### Parameter selection for evolutionary algorithm

**Figure 1:**
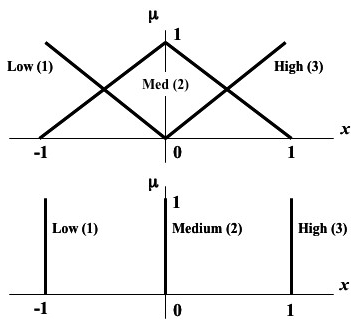
Schematic of fuzzification (triangular sets; above) and defuzzification (point-centroid; below), where *x *is the normalized ratiometric data point (i.e., the arctangent divided by π/2 of the base 2 logarithm of gene expression ratio) and μ is the fuzzy set membership function.

**Figure 2:**
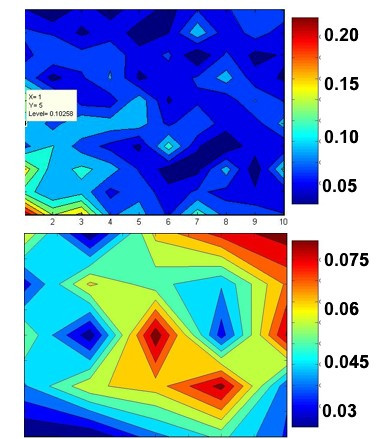
Sample contour plots for the errors (*E*, in the range as shown in the accompanying legend) where mutation probability is plotted against the crossover probability. The top plot is for *p*_*C *_(horizontal axis) and *p*_*M *_(vertical axis) from 0.1 to 1.0, and the bottom is for *p*_*C *_and *p*_*M *_greater than 0.6.

As described in the Methods, we analyzed artificial data to choose suitable parameters for the evolutionary algorithm: the number of iterations ("generations"), the number of rule combinations generated and evaluated ("population size"; *N*), and the probabilities of mutation and crossover (*p*_*M *_and *p*_*C*_, respectively). Focusing on *p*_*M *_and *p*_*C *_first, we generated artificial data for *G *= 30 genes and, as outlined in the Methods, aimed to find a rule set with a minimum error value (approaching zero) for the 30th gene. Gene expression data were fuzzified using the scheme described in the Methods as shown in Figure 1, and rules were applied as in Table 1. The algorithms were run for 50 generations and a population size of *N *= 50 generations. The multiple runs used different seeds for pseudorandom number generation to avoid a "capitalization by chance" bias. These parameters were chosen for computational tractability. Extensive parameter analysis revealed that *p*_*M *_and *p*_*C *_less than 0.6 can result in the algorithm falling into local minima and failing to find rule sets with *E *< 0.1 in many cases. Figure 2 displays sample *p*_*M *_versus *p*_*C *_contour plots, showing that above 0.6, the errors are acceptably bound. We identified *p*_*C *_and *p*_*M *_equal to 0.7 for robust algorithm performance (mean *E *= 0.015 on artificial data over 500 runs and ability to find a best-fitting rule set with *E *= 0 for G = 10 genes).

**Table 1:** Fuzzy Rules Allowed at a Network Node.

**Rule #**	**If input is...**	**Then output is...**	**Rule #**	**If input Is...**	**Then output is...**
	Low	High		Low	Low
-3	Medium	Medium	1	Medium	Medium
	High	Low		High	Medium

	Low	High		Low	Medium
-2	Medium	Medium	2	Medium	Medium
	High	Medium		High	High

	Low	Medium		Low	Low
-1	Medium	Medium	3	Medium	Medium
	High	Low		High	High

**Figure 3:**
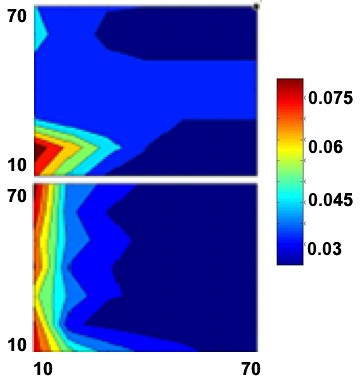
Sample contour plots for the errors (*E*, in the range as shown in the accompanying legend) where the population size (number of rule sets tested in each generation) is plotted against the number of generations (algorithm iterations). The top plot is for 10 genes and the bottom is for 100 genes.

Next, we identified the optimal population size (*N*) and number of generations (algorithm iterations) using a similar procedure, ensuring that our choice was relatively insensitive to the number of genes in our target range (*G *up to 100). As shown in the sample contour plots in Figure 3, effective results are found for relatively low numbers of rule sets in the population and algorithm generations. Thus, we set each parameter at 30. (Data not shown verified that this choice does not affect our determination of mutation and crossover probabilities.) Overall, for our choices of parameters, we determined that the algorithm takes approximately 40 minutes to run with Matlab 7.0 R14 on a PC laptop with an Intel Pentium M processor at 1.80 GHz.

### Human cell cycle gene data set

The dataset used for optimization and testing of the GA method is a published experiment [18] conducted to characterize the genome-wide transcriptional program of the cell division cycle in mammalian cells from a human cancer cell line (HeLa) using cDNA microarrays. The HeLa cells were synchronized using three methods: (1) a thymidine block arresting them in S phase (datasets labeled with the prefix "TT"), (2) a thymidine-nocodazole block arresting them in mitosis (datasets labeled with the prefix "TN"), and (3) a physical method, mitotic shake-off (dataset labeled "Shake").

Table 2 shows the 20 genes that were selected in the original paper [18] as "cell cycle marker genes" since they have been identified as being cell cycle-regulated by previous biological knowledge. These genes are significant in different phases of the cell cycle (as shown in Table 2). These genes have known regulatory relationships; consequently, their behavior in the Whitfield et al. [18] data sets can be readily interpreted biologically. Thus, we use the human cell cycle marker gene set listed in Table 2 to validate the methods for model generation presented here.

**Table 2:** Human Cell Cycle Marker Gene Set

	***Phase***	***Gene Name***
1	G1/S	CCNE1
2	G1/S	E2F1
3	G1/S	CDC6
4	G1/S	PCNA

5	S	RFC4
6	S	DHFR
7	S	RRM2
8	S	RAD51

9	G2	CDC2
10	G2	TOP2A
11	G2	CCNF
12	G2	CCNA2

13	M	STK15
14	M	BUB1
15	M	CCNB1
16	M	PLK1

17	M/G1	PTTG1
18	M/G1	RAD21
19	M/G1	VEGFC
**20**	M/G1	CDKN3

### Parameter estimation for error probability distribution function

**Figure 4:**
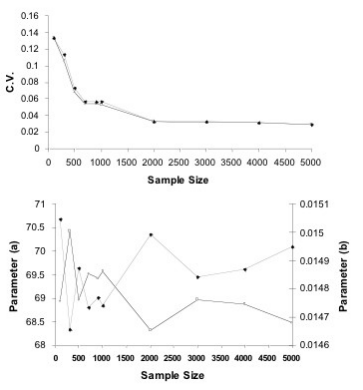
Estimation of gamma function parameters for the error probability distribution of gene TOP2A, with the TN data set. (Bottom) The mean *a *and *b *parameters (solid line with white squares and grey line with black diamonds, respectively) estimated for increasing sample sizes uniformly drawn from the space of all possible rule sets. (Top) Coefficients of variation (standard deviation divided by mean) versus sample size (based on 10 samplings).

As described in the Methods section, we randomly sample rules to estimate the gamma probability distribution parameters for the probability that the algorithm finds a rule with a given error *E *or lower by chance. Different data sets for different genes will generate a different probability distribution. The shape of the probability of distribution will change as a consequence of the level of noise and the amount of correlation between genes in a particular data set. We first determined a robust number of rules to randomly select for evaluation to fit the distribution. Figure 4 shows the value for the gamma distribution parameter and the coefficients of variation of the estimates for *a *and *b *(Equation 4) for increasing sample sizes, providing the gene TOP2A and TN1 data set as an example. Based on analysis of other genes, we conservatively chose to sample 2000 rules.

**Figure 5:**
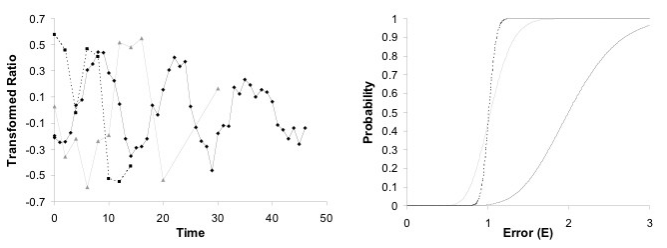
Time series data (left) and corresponding error distribution functions for specific genes: the solid line is data set TT3 for gene CCNB1 (*a *= 22.8, *b *= 0.0458, *a*/*b *= 498), the grey line is TT1 for CCNE1 (*a *= 9.3, *b *= 0.0120, *a*/*b *= 77), and the dashed line is Shake for CDKN3 (*a *= 197, *b *= 0.00517, *a*/*b *= 38 092).

Figure 5 shows the computed error distribution function for three selected genes with data from three different data sets (Figure 5 also shows the corresponding time series data). Notably, each gene has a different ratio of gamma parameters *a*/*b*, which lead to a sharp step-like transition (large *a*/*b*) through to a gentle slope (small *a*/*b*). Table 3 shows the ratio *a*/*b *divided by 1000 for each gene in the human cell cycle marker gene set (from Table 2) and the average ratio for each data set.

**Table 3:** Ratio of error distribution function parameters (*a*/*b*)

Gene	TT1	TT2	TT3	TN	Shake
CCNE1	0.498	0.825	0.292	0.291	0.623
E2F1	1.431	0.622	0.293	1.460	0.604
CDC6	0.082	0.234	0.307	0.366	1.320
PCNA	0.917	0.283	0.320	2.303	1.107
RFC4	0.137	0.251	0.058	0.200	0.540
DHFR	0.701	0.147	0.148	0.057	0.029
RRM2	2.013	1.607	3.081	0.481	0.541
RAD51	0.513	0.955	0.400	0.120	2.025
CDC2	0.677	1.612	3.407	6.814	0.304
TOP2A	0.567	0.496	0.931	4.315	0.691
CCNF	0.402	0.944	0.678	0.423	2.468
CCNA2	0.350	0.293	0.320	0.557	0.822
STK15	1.108	1.285	0.866	1.644	3.346
BUB1	0.285	0.405	0.231	0.444	0.185
CCNB1	0.249	0.717	0.078	0.181	0.268
PLK1	0.899	1.081	1.234	0.851	3.003
PTTG1	0.289	0.376	0.244	0.447	1.993
RAD21	0.093	0.106	0.141	0.334	0.029
VEGFC	0.032	0.252	0.130	0.200	0.523
CDKN3	0.252	0.419	0.177	0.679	38.093

**Average**	**0.57**	**0.65**	**0.67**	**1.11**	**2.93**

### Identification and analysis of fuzzy network models

**Figure 6:**
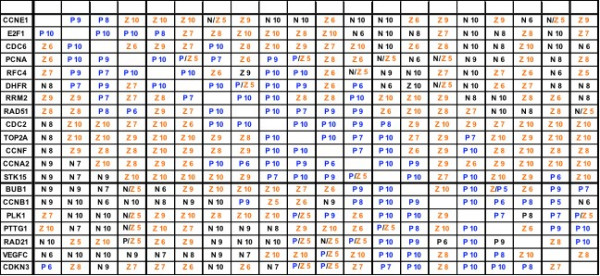
Rules obtained from the TT3 data set for genes listed in Table 2. Outputs are in columns in the same sequence as the labeled inputs in rows. The majority of rules (zero, positive or negative based on the rule definitions in Table 1) are given (Z, P, and N respectively) along with the number of rule sets (out of 10) with that rule. In the case of a tie, 5 each, both are given in the appropriate cell.

The first data set from [18] we use to develop fuzzy network models (the "training set") for the cell cycle marker genes is "TT3", which has data for the most time points. As illustrated in Table 4, for each gene we obtain multiple rule sets on runs of the evolutionary algorithm with a different random number generator seed. Each of these represents the lowest error for a member of the rule set population in the final iteration of the algorithm, and they have similarly low probabilities of being found at random (based on the calculation as discussed above). Supplementary Table 1, Additional File 1 shows all of the results for all of the genes. Figure 6 summarizes the results of ten algorithm runs using the TT3 data set to identify rules for all 20 genes in Table 2. It shows the number of "positive" rules (P) which are defined as **+1**, +**2**, and +**3 **in Table 1, "negative" rules (N; **-1**, **-2**, and **-3**), and zero rules indicating that the corresponding input genes make no contribution.

**Table 4:** Rules for CCNE1 and Training Errors for TT3 Data Set

**Rules for input genes in columns corresponding to rows in Table 2**																				**ERROR**	**P(E)**
0	3	0	0	0	0	-3	0	-3	-3	-3	-3	-3	-3	0	-3	0	-3	-3	0	0.158	1.57E-09
0	3	3	0	0	0	-3	0	-3	-3	0	-3	-3	0	0	-3	0	-3	0	0	0.139	2.27E-10
0	3	3	0	0	0	0	0	-3	-3	0	-3	-3	-2	0	-3	0	0	0	1	0.152	8.78E-10
0	3	0	0	0	0	0	-1	-3	-3	0	-3	-3	-2	0	-3	0	-3	-3	0	0.166	3.27E-09
0	3	3	0	0	0	-3	0	-3	-3	0	-3	-3	0	0	-3	0	0	-3	0	0.146	4.78E-10
0	0	3	0	0	0	-3	0	-3	-3	-3	-3	-3	-1	0	-3	0	-3	-3	0	0.168	3.91E-09
0	3	2	0	0	0	0	0	-3	-3	-1	-3	-3	0	0	-3	0	-3	0	0	0.158	1.57E-09
0	2	0	0	0	0	0	0	-3	-3	0	-3	-3	0	0	-3	0	-1	0	0	0.142	3.14E-10
0	3	3	0	0	0	-3	0	-3	-3	-1	-3	-3	0	0	-3	0	0	-2	0	0.162	2.28E-09
0	3	3	0	0	0	0	0	-3	-3	0	-3	-3	0	-3	-3	1	0	0	0	0.154	1.07E-09

**Figure 7:**
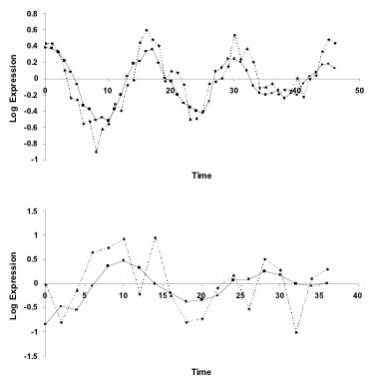
Plots of experimental [18] and simulated data for the PCNA gene using the fuzzy rule set model found for the TT3 training data set. Shown here are (top) a typically reasonable fit, *E *= 0.249 and *P *= 3.47*10^-7 ^on the training data set, and (bottom) a typically poorer fit (for a rule found using the evolutionary algorithm), *E *= 0.745 and *P *= 1.37*10^-2 ^on the TN data set. The plots show the base 2 logarithm of the gene expression ratios (so the simulated results are transformed back from the normalized form) versus points in the experimental time series (arbitary units).

As Table 4 illustrates, in most cases a majority or "consensus" rule associating an input gene to an output gene can be identified. Supplementary Table 2, Additional File 1 shows the "best" (minimum error) rule sets found for each gene, along with "consensus" rule sets: these are combinations of the rule found for each input gene in a plurality of cases (there are two consensus rule sets generated by ties – no more than two were found for any given gene). For example, referring to Table 4, the consensus rule for the dependence of CCNE1 on E2F1 (the first column of Table 4) is **3**. Supplementary Table 2, Additional File 1 also shows consensus rule sets (in this case the one with the lowest error is included) obtained by using the TT2 and Shake data sets as alternative training sets instead of TT3. Figure 7 shows sample plots of predicted and experimental [18] data for the best rules found using the TT3 training set on both the original TT3 data set as well as the TN data set.

Supplementary Table 3, Additional File 1 has the errors and probability of finding by chance for each gene for the test data sets, using TT3 and alternate training sets. Table 5 is an excerpt of Supplementary Table 3, Additional File 1 focusing on the prediction of gene data in the TT3 data set, using the minimum error ("best") rule set obtained using TT3 itself as training data, as well as errors and probability of finding a rule set by chance using other data sets (TT2 and Shake) as training data.

**Table 5:** Evaluating Rule Sets on TT3 Data

	**TT3**		**TT2**		**Shake**	
	**Error**	**P(E)**	**Error**	**P(E)**	**Error**	**P(E)**
**CCNE1**	0.139	2.18E-10	0.186	1.79E-08	0.192	2.67E-08
**E2F1**	0.143	2.71E-10	0.224	2.08E-07	0.240	5.29E-07
**CDC6**	0.083	2.55E-14	0.187	1.00E-08	0.518	4.61E-03
**PCNA**	0.249	3.47E-07	0.437	5.56E-04	0.518	3.57E-03
**RFC4**	0.130	3.85E-06	0.588	5.47E-02	1.293	6.78E-01
**DHFR**	0.230	4.15E-06	0.418	1.37E-03	1.161	5.65E-01
**RRM2**	0.275	2.16E-16	0.412	1.28E-09	0.705	3.85E-03
**RAD51**	0.220	4.83E-09	0.295	5.76E-07	0.462	3.58E-04
**CDC2**	0.299	3.39E-15	0.302	5.20E-15	0.489	2.97E-07
**TOP2A**	0.073	7.26E-25	0.100	4.91E-21	0.383	1.62E-06
**CCNF**	0.117	1.56E-16	0.169	6.65E-13	0.559	2.73E-03
**CCNA2**	0.142	1.15E-10	0.208	4.49E-08	0.241	3.73E-07
**STK15**	0.089	2.06E-21	0.172	6.73E-14	0.220	2.47E-11
**BUB1**	0.097	1.02E-11	0.124	3.46E-10	0.113	8.71E-11
**CCNB1**	0.368	2.94E-03	1.307	7.24E-01	0.683	1.00E-01
**PLK1**	0.153	5.23E-18	0.261	2.54E-11	0.264	3.41E-11
**PTTG1**	0.150	2.38E-09	0.180	3.08E-08	0.404	5.83E-04
**RAD21**	0.278	5.76E-05	0.474	6.08E-03	0.515	1.12E-02
**VEGFC**	0.110	1.27E-08	0.163	7.62E-07	0.191	3.77E-06
**CDKN3**	0.220	2.92E-06	0.295	6.53E-05	1.836	9.90E-01

### Convergence of evolutionary algorithm to exhaustive search results

We used exhaustive search (as was done in our previous work [11]) to generate rules for a subset of cell cycle genes and verify convergence of the evolutionary search employing the algorithm parameters determined above. In this case, we selected ten genes and used the "TT3" data set to determine the rules, as shown in Table 6. We constrained the space of potential rules to **-3**, **0**, and **3**, though we did allow any of the 9 other genes to be an input of the output gene being calculated. The constraints on the number of genes and possible interaction rules were designed to preserve computational resources. The exhaustive search ran in approximately 2.5 hours using Matlab 7.0 R14 on a PC laptop with an Intel Pentium M processor at 1.80 GHz. Under the same constraints, the evolutionary search was completed (for all 10 genes) in 260 seconds, with mutation and crossover probabilities set at 0.7, population size of 30, and 50 generations. We ran the algorithm 10 times with distinct seeds, and determined the generation at which the best-fitting rule in the population matched the result of the exhaustive search. Table 6 shows the average and maximum number of generations at which convergence occurred. In two instances (out of 100 total, 10 genes and 10 runs), the evolutionary method failed to reach the exhaustive search solution. In both cases this was for the output gene E2F1, and the same local minimum was found, a rule with error of 0.134 (compared with 0.117). Notably, in each of the other 8 trials, this rule appeared as the best fit at an early stage prior to convergence to the global minimum.

**Table 6:** Exhaustive Search Results and Evolutionary Method Evaluation

	**Outputs**									
**Inputs**	BRCA1	CDC25B	E2F1	CDC2	CDKN1B	CCNA2	POLD3	PCNA	GADD45A	MDM2
BRCA1	0	0	0	0	-3	0	0	0	0	0
CDC25B	0	0	-3	0	0	3	0	0	0	3
E2F1	3	-3	0	0	0	-3	3	3	-3	0
CDC2	3	0	0	0	3	0	0	0	0	-3
CDKN1B	-3	0	-3	3	0	0	0	0	3	3
CCNA2	0	3	0	3	3	0	0	0	0	3
POLD3	0	-3	3	0	0	0	0	3	0	-3
PCNA	0	0	3	0	3	0	3	0	0	0
GADD45A	-3	0	0	0	3	0	0	0	0	-3
MDM2	-3	3	0	0	0	0	-3	0	-3	0

**Fit Error (E)**	0.431	0.283	0.117	0.432	0.219	0.222	0.221	0.323	0.508	0.49
**Ave. # Gen.**	16.5	12.8	13.5	12.5	9.7	10.1	11	14.2	12.8	14.6
**Max. # Gen.**	34	18	*	17	14	15	19	23	21	20

* For E2F1, in two cases the global minimum error rule set was not found after 50 generations due to convergence to a local minimum with a higher fit error (0.134).

## Discussions and conclusion

In this paper, we describe a computationally tractable method to identify fuzzy logic-based models for biomolecular networks (genes, proteins, etc.) from semi-quantitative data sets generated by high-throughput experiments. We demonstrate and validate the method to identify a 20-gene cell cycle model using published sets of gene expression microarray data. As described in our previous work [11] and illustrated in Figure 7, fuzzy logic models have sufficient resolution and precision to quantitatively fit measurements. The key advances presented here are the simplification of the rule scheme (as shown in Table 1), application of an evolutionary algorithm to identify the best-fitting rule network models, and the development of a probability model to evaluate the performance of rule identification.

Based on our analysis of simulated data, the parameters of the evolutionary algorithm lead to a scalable method for potentially as many as 150 variables. As shown in Figure 3, algorithm performance is robust with only 30–50 iterations (and the same number of solutions evaluated per iteration). The algorithm does require a relatively high crossover and mutation probability (as shown in Figure 2). Notably, our approach here is to use a simple formulation of the mutation and crossover operations of the evolutionary algorithm. We are currently exploring more sophisticated operators that will function at lower probabilities, which will improve algorithm performance (by reducing the probability of having to do the searching and manipulation required to execute the operator). Even in its current state, the evolutionary method can converge to the global minimum error rule set identified by exhaustive search, as shown in Table 6, with a very low probability of being trapped in a local minimum. The latter analysis exemplifies the primary benefit of the methodology presented in this paper: the evolutionary method runs in only a few minutes where the exhaustive search took hours, even for constrained rule spaces and numbers of potential input genes.

The gamma distribution-based probability function we introduce in this publication allows us to evaluate search performance. With it, we can estimate the probability of finding a rule with an equal or better fit error than the outcome of the search algorithm. As an exhaustive search for the rules would show, there are in general multiple models fitting the data equally well. In general, this depends on the nature of the data set that is being used to identify models. As Table 3 shows, the cell cycle data set synchronized using the mechanical "Shake-Off" method has a gamma distribution fit over all the genes that leads to the greatest redundancy with overall higher fit errors (as illustrated in Figure 5). This correlates with the conclusion from the original experimental publication [18] that the "Shake" synchronization method is less effective and consistent than chemical stimuli (i.e., as used for the TT data sets). Furthermore, as shown in Table 5, models identified using the "Shake" data set are less effective in predicting data from another set.

The fuzzy rule models identified using the TT3 training data set are capable of qualitatively predicting cell cycle gene network function. Figure 6 displays cell cycle genes listed in the same order as Table 2. By inspection, positive interaction rules are generally found for genes active in the same phase of the cell cycle network, and negative interaction rules are generally found for genes in opposite phases of the cell cycle network. Notably, multiple rule sets found for each gene interaction show similar results.

It is critical to appreciate that given the noise levels in biological experiments, multiple models will be consistent with the data. These models represent hypotheses, which can be constrained by prior knowledge. Modeling redundancy is a key reason why we have designed our method to be scalable for on the order of 10^2 ^variables (proteins and/or genes). This is because capitalization by chance will occur for larger potential solution spaces, leading to a huge number of candidate hypothetical models consistent with data sets, which are impossible to interpret. As shown by our results, such as those in Table 4, we can optimally obtain 2–3 rule models for each gene on multiple runs of the evolutionary algorithm, which represents a constrained set of potential hypotheses. Then, the subsequent round of experiments can be optimally designed based on the hypotheses identified from the first round of model identification, leading to further constraints and eventually better understanding of biological system functions.

One key requirement of the method presented here is that data be ratiometric, due to the definition of fuzzy sets. This can be a drawback, since Affymetrix gene expression experiments, as well as mass spectrometry in proteomic and metabolomic studies, do not generate data in the form of ratios. While it would be possible to redefine fuzzy sets for other kinds of data, such a solution would sacrifice the robustness and generality of the approach. In many cases, this is not a major problem: there is a control measurement, or a pooled sample that is used as a reference to generate ratios of expression values. Alternatively, expression values can be normalized to a set of standards on the chip, or to a global mean. In the future, we will develop formal means of analyzing non-ratiometric data encountered in proteomics and metabolomics.

Comprehensive systems biology models of cellular function require integrating representations both the regulation and activities of proteins. Gene and protein regulation involve logical switches, and it is generally less sensitive to quantitative parameters over a wide range as long as the qualitative behavior remains stable [19-21]. There is also a high degree of complexity, with hundreds genes possibly changing expression during a stress condition (as seen with the cell cycle data set [18]). Consequently, scalable, generally qualitative or logical network analysis is appropriate. Quantitative models are overkill, and are unsuitable for fitting models based on data: for biomolecular networks, establishing connectivity and activation/inhibition relationships are far more important than estimating parameter values. On the other hand, biochemical pathways catalyzed by the enzymatic function of proteins, for example, are well-suited for quantitative modeling based on chemical kinetics. Fuzzy logic is a framework that can bring together physics-based models with more logical regulatory models, building a foundation for multiscale biomolecular network models. Unlike Bayesian or other hybrid modeling approaches, fuzzy logic has the additional advantage of an intuitive connection to linguistic and graphical models of biomolecular networks that are present in the biological literature. In parallel with the work shown here, we are developing methods for integrating scalable fuzzy logic models with the results of large scale data-mining from genome databases (i.e., gene ontologies such as GO annotations) and text sources (i.e., NIH PubMed) [22].

## Methods

### Fuzzy logic representation of data

The method used for fuzzy logic network models is based on that presented in our earlier work [11]. As before, the biomolecular networks analyzed in this paper consist entirely of genes, and the data are gene expression ratios from microarray experiments. However, in principle the method is general for any kind of network node or data type, though the *fuzzification *(conversion from number to fuzzy set membership) algorithm is designed for ratiometric data. The basic concept of fuzzy logic is that quantities are defined by a membership function from 0.0 to 1.0 in one or more fuzzy sets, where 0 corresponds to Boolean False and 1 to True, with a continuum between. We define three fuzzy sets for gene expression: Low (designated as **1**), Medium (**2**), and High (**3**). The set Low is for expression ratios less than 1 (underexpression) and the set High corresponds to overexpression (ratio greater than 1). Gene expression ratios obtained from experimental data are converted to base 2 logarithms (to make them symmetric). To normalize the values on a range from -1.0 to 1.0 centered at 0, we take the arctangent and divide by π/2 as described in [11]. Membership functions *y*_1_, *y*_2_, and *y*_3 _in sets Low, Medium, and High respectively are defined from the normalized logarithm of the gene expression ratio *x *as

y1={−xx<00x>0y2=1−|x|y3=={0x<0xx>0
 MathType@MTEF@5@5@+=feaafiart1ev1aaatCvAUfKttLearuWrP9MDH5MBPbIqV92AaeXatLxBI9gBaebbnrfifHhDYfgasaacH8akY=wiFfYdH8Gipec8Eeeu0xXdbba9frFj0=OqFfea0dXdd9vqai=hGuQ8kuc9pgc9s8qqaq=dirpe0xb9q8qiLsFr0=vr0=vr0dc8meaabaqaciaacaGaaeqabaqabeGadaaakqaabeqaaiabdMha5naaBaaaleaacqaIXaqmaeqaaOGaeyypa0ZaaiqaaeaafaqabeGacaaabaGaeyOeI0IaemiEaGhabaGaemiEaGNaeyipaWJaeGimaadabaGaeGimaadabaGaemiEaGNaeyOpa4JaeGimaadaaaGaay5EaaaabaGaemyEaK3aaSbaaSqaaiabikdaYaqabaGccqGH9aqpcqaIXaqmcqGHsisldaabdaqaaiabdIha4bGaay5bSlaawIa7aaqaaiabdMha5naaBaaaleaacqaIZaWmaeqaaOGaeyypa0Jaeyypa0ZaaiqaaeaafaqabeGacaaabaGaeGimaadabaGaemiEaGNaeyipaWJaeGimaadabaGaemiEaGhabaGaemiEaGNaeyOpa4JaeGimaadaaaGaay5Eaaaaaaa@54F0@

Equation 1 defines the fuzzification of variables using triangular fuzzy sets, as shown in Figure 1. The fuzzified quantity is converted back into an absolute number x˜
 MathType@MTEF@5@5@+=feaafiart1ev1aaatCvAUfKttLearuWrP9MDH5MBPbIqV92AaeXatLxBI9gBaebbnrfifHhDYfgasaacH8akY=wiFfYdH8Gipec8Eeeu0xXdbba9frFj0=OqFfea0dXdd9vqai=hGuQ8kuc9pgc9s8qqaq=dirpe0xb9q8qiLsFr0=vr0=vr0dc8meaabaqaciaacaGaaeqabaqabeGadaaakeaacuWG4baEgaacaaaa@2E34@ using the centroid point defuzzification defined by

x˜=y3−y1y1+y2+y3
 MathType@MTEF@5@5@+=feaafiart1ev1aaatCvAUfKttLearuWrP9MDH5MBPbIqV92AaeXatLxBI9gBaebbnrfifHhDYfgasaacH8akY=wiFfYdH8Gipec8Eeeu0xXdbba9frFj0=OqFfea0dXdd9vqai=hGuQ8kuc9pgc9s8qqaq=dirpe0xb9q8qiLsFr0=vr0=vr0dc8meaabaqaciaacaGaaeqabaqabeGadaaakeaacuWG4baEgaacaiabg2da9maalaaabaGaemyEaK3aaSbaaSqaaiabiodaZaqabaGccqGHsislcqWG5bqEdaWgaaWcbaGaeGymaedabeaaaOqaaiabdMha5naaBaaaleaacqaIXaqmaeqaaOGaey4kaSIaemyEaK3aaSbaaSqaaiabikdaYaqabaGccqGHRaWkcqWG5bqEdaWgaaWcbaGaeG4mamdabeaaaaaaaa@3F20@

As described in our previous work, the choices of fuzzy set structure and fuzzification scheme were made to maximize computational efficiency and avoid introducing systematic error in the fuzzy approximation.

### Fuzzy rule-based network models

The rules for interactions in the biomolecular network model are defined based on the fuzzified expression state of the network nodes (in this case, genes). The rules are defined linguistically, i.e., "If Gene A is Low then Gene B is High". Under the current implementation, each rule is applied for all data points in a series of experiments (e.g., a time series). The state-based rule is evaluated at each point independently; thus, there are no time delays. (Time delays in gene expression are difficult to implement due to the imprecision in defining "transcription time" and more significantly, accurately modeling the time required for a transcription-level change to be relevant for regulation.) To make the method more computationally tractable, we only allow 7 of the 27 possible rule combinations for how the input A affects the fuzzy state of the node (output) B. As shown in Table 1, these rules are designated **-3 **through **+3**, where positive rules represent a positive correlation and vice versa. The rule **0 **represents the case when there is no rule ("null").

As in our previous work, we use a linearized fuzzy rule configuration (following [14]). The rules for the multiple inputs to a node are evaluated separately based on the state of those outputs, and the fuzzy set membership functions so obtained are summed (implementing the fuzzy set operation equivalent to Boolean OR). Unlike our previous work, we do *not *limit the number of possible inputs to a node. However, the implementation does allow the user to specify which of the 7 possible rules can be applied for each input-output combination. Specifying only the null rule **0 **for a gene effectively eliminates it as a possible input. Defining what rules are allowed (e.g., eliminating an input, only allowing positive rules, etc.) is a means of introducing prior biological knowledge to limit the search space for plausible models. (In the examples shown in this paper, there are no such limits imposed on the search.)

### Evolutionary search algorithm

In our previous work on fuzzy network model identification [11], we employed an exhaustive search for all possible network models and then compared the prediction error versus a training data set to rank the rules by their prediction error. Now, we have developed an evolutionary algorithm to accelerate the search for the most plausible sets of rule combinations defining a biomolecular network model. *Plausibility *is defined by the minimum value of *E *obtained using Equation 3 for a training data set. Genetic algorithms [23] are inspired by natural selection in evolution, in which the fittest individuals in a generation pass on their genes, with probabilistic recombination and mutation. Because of the linearization, each node is computed independently, so the algorithm runs for each node sequentially without depending on previous results. Our method consists of the following steps, considering a particular node (e.g., gene) as the "output".

#### 1. Generation of initial population

The user can specify which rules are allowed for each possible input-output combination for the *G *nodes (e.g., genes) in the biomolecular network. Based on that possible space, *N *rule combinations are generated for the output. Each rule is a string of *G *randomly selected rules (numbered as in Table 1) for each input in sequence.

#### 2. Evaluation of the fitness function

The "fitness" of each of the *N *rule combinations is calculated using the normalized sum-of-squares error function *E *defined by Equation 3. Maximum fitness is determined by minimum *E*.

#### 3. Reproduction, crossover, mutation

A duplicate set of *N *rule combinations are generated as the "New Population". Within this New Population, crossover and mutation are performed. All crossovers are performed before the mutations. In crossover, there is a probability *p*_*C *_that each of the new rule combinations will be selected for a crossover. If one is selected, then another member of the New Population is identified (from an unweighted, uniform distribution). Then, a random number of rules in the same points in the sequence of each of the two partner rule combinations (corresponding to rules for particular inputs) are selected and the rules in them are switched. (If the rules crossing over are the same, then the recombination has no effect.) In mutation, there is a probability *p*_*M *_for each of the *N *new rule combinations to have a single mutation. If a mutation occurs, it occurs for a (uniformly distributed) random number of the inputs in the rule combination, and then another rule (circumscribed by the user-imposed constraint in Step 1) is randomly selected for each of the inputs selected for a mutation.

#### 4. Selection of the next generation

The fitness function (*E*) is calculated for each rule combination in the New Population. Then, the *N *most fit (least *E*) rule combinations from the 2*N *total in the Old and New Populations are selected for the next generation, repeating from step 3.

#### 5. Termination

The search is generally terminated when either the minimum *E *within the total population changes by less than a certain threshold, or more commonly in our implementation by attaining a certain number of generations. This is because we view this as a means of identifying plausible network models consistent with data rather than the absolute lowest *E*, which is affected by the high levels of noise in microarray and other high-throughput biological data.

As shown in our results, we suggest that the parameters of the evolutionary algorithm should range from 0.6 to 0.7 for both *p*_*C *_and *p*_*M*_, and *N *from 30 to 50 for typical gene expression data, with 30–50 generations giving stable termination at a consistently close-to-minimum *E *for the final population of rule combinations. These parameters were chosen using an artificial data set with *G *genes (nodes) and *M *data points for each gene. The data are *M *randomly generated expression values for each of (*G*-1) genes, and then a *G*th gene for which the values are created based on a randomly generated rule set with the other (*G*-1) genes as possible inputs. Notably, unless *M *> *G*, there will be multiple rule sets that can specify the *G*th gene, and even then, because of the limited granularity of fuzzy sets there can still be multiple solutions. Consequently, the parameters were chosen to consistently find *E *≈ 0 rather than the particular rule set used to generate the artificial data.

### Rule discovery probability function

The defuzzified (using Equation 2) predicted expression ratios {x˜i}
 MathType@MTEF@5@5@+=feaafiart1ev1aaatCvAUfKttLearuWrP9MDH5MBPbIqV92AaeXatLxBI9gBaebbnrfifHhDYfgasaacH8akY=wiFfYdH8Gipec8Eeeu0xXdbba9frFj0=OqFfea0dXdd9vqai=hGuQ8kuc9pgc9s8qqaq=dirpe0xb9q8qiLsFr0=vr0=vr0dc8meaabaqaciaacaGaaeqabaqabeGadaaakeaadaGadaqaaiqbdIha4zaaiaWaaSbaaSqaaiabdMgaPbqabaaakiaawUhacaGL9baaaaa@31F6@ of the output (node) gene is compared to the experimental data series {*x*_*i*_} in a similar fashion to our previous work, using the sum of squares error expression

E=∑j=1M(xi−x˜i)2∑j=1M(xi−x¯)2
 MathType@MTEF@5@5@+=feaafiart1ev1aaatCvAUfKttLearuWrP9MDH5MBPbIqV92AaeXatLxBI9gBaebbnrfifHhDYfgasaacH8akY=wiFfYdH8Gipec8Eeeu0xXdbba9frFj0=OqFfea0dXdd9vqai=hGuQ8kuc9pgc9s8qqaq=dirpe0xb9q8qiLsFr0=vr0=vr0dc8meaabaqaciaacaGaaeqabaqabeGadaaakeaacqWGfbqrcqGH9aqpdaWcaaqaamaaqahabaWaaeWaaeaacqWG4baEdaWgaaWcbaGaemyAaKgabeaakiabgkHiTiqbdIha4zaaiaWaaSbaaSqaaiabdMgaPbqabaaakiaawIcacaGLPaaadaahaaWcbeqaaiabikdaYaaaaeaacqWGQbGAcqGH9aqpcqaIXaqmaeaacqWGnbqta0GaeyyeIuoaaOqaamaaqahabaWaaeWaaeaacqWG4baEdaWgaaWcbaGaemyAaKgabeaakiabgkHiTiqbdIha4zaaraaacaGLOaGaayzkaaWaaWbaaSqabeaacqaIYaGmaaaabaGaemOAaOMaeyypa0JaeGymaedabaGaemyta0eaniabggHiLdaaaaaa@4E21@

In Equation 3, there are *M *data points in the series and x¯
 MathType@MTEF@5@5@+=feaafiart1ev1aaatCvAUfKttLearuWrP9MDH5MBPbIqV92AaeXatLxBI9gBaebbnrfifHhDYfgasaacH8akY=wiFfYdH8Gipec8Eeeu0xXdbba9frFj0=OqFfea0dXdd9vqai=hGuQ8kuc9pgc9s8qqaq=dirpe0xb9q8qiLsFr0=vr0=vr0dc8meaabaqaciaacaGaaeqabaqabeGadaaakeaacuWG4baEgaqeaaaa@2E3D@ is the average expression ratio over the whole series. The denominator of Equation 3 is used to "normalize" the error to compare genes with high and low expression ratio magnitudes.

Because in general *M *must be significantly greater than *G *(the number of inputs to a node) to specifically define a particular optimal (minimum *E*) rule combination, there are generally multiple redundant solutions for any given value of *E*. Indeed, even if there were specified optimal solutions, this redundancy would likely still exist because of the limited granularity of the fuzzy logic approximation defined by Equations 1 and 2. Consequently, if the evolutionary search finds a particular rule with an error *E*, it is important to compute the probability *p*_E_(*E*, *D*) of simply randomly selecting a rule out of the 7^*G *^possibilities in the search space that has that value of *E *or lower (i.e., the probability of finding that rule by chance). In general, this will depend on the data matrix *D *for the node (i.e, the time series of expression ratios for the input genes and the gene to which a rule combination is being fit).

As seen in our previous work doing exhaustive searches that generate all possible rule combinations, the histogram for *E *is roughly bell-shaped (but skewed since it is a ratio): there are a few rule combinations with a low *E *corresponding to "good fits", a large number with a moderate *E *that are poor fits, and a small number that represent anti-correlations, leading to a sigmoidal cumulative distribution [11]. Based on our previous results and keeping *E *strictly positive, we propose to estimate the error distribution through a gamma distribution, which is defined using two parameters, *a *and *b *as,

y=f(x|a,b)=1baΓ(a)xa−1e−x/b
 MathType@MTEF@5@5@+=feaafiart1ev1aaatCvAUfKttLearuWrP9MDH5MBPbIqV92AaeXatLxBI9gBaebbnrfifHhDYfgasaacH8akY=wiFfYdH8Gipec8Eeeu0xXdbba9frFj0=OqFfea0dXdd9vqai=hGuQ8kuc9pgc9s8qqaq=dirpe0xb9q8qiLsFr0=vr0=vr0dc8meaabaqaciaacaGaaeqabaqabeGadaaakeaacqWG5bqEcqGH9aqpcqWGMbGzdaqadaqaaiabdIha4naaeeaabaGaemyyaeMaeiilaWIaemOyaigacaGLhWoaaiaawIcacaGLPaaacqGH9aqpdaWcaaqaaiabigdaXaqaaiabdkgaInaaCaaaleqabaGaemyyaegaaOGaeu4KdC0aaeWaaeaacqWGHbqyaiaawIcacaGLPaaaaaGaemiEaG3aaWbaaSqabeaacqWGHbqycqGHsislcqaIXaqmaaGccqWGLbqzdaahaaWcbeqaaiabgkHiTmaalyaabaGaemiEaGhabaGaemOyaigaaaaaaaa@4BC2@

To obtain a maximum likelihood estimate of *a *and *b *for a particular variable in a given data set, we evaluate *E *using Equation 3 for a random sample of the search space of rule combinations for that variable in that data set. Based on our results (reported in the Results), for *G *= 20 nodes (genes) in the network, we have found that on the order of 10^2 ^rule combinations (of the 7^20 ^possibilities) need to be evaluated for a particular data set to obtain a stable parameter estimate.

### Software implementation

All of the computational methods described in the Methods, including fuzzification, error determination, the evolutionary algorithm, artificial data set generation, and rule probability function evaluation are implemented in Matlab 7.0 Release 14 (The Mathworks, Inc., Natick, MA, USA). All M-files are available from the corresponding author upon request. Gamma distribution routines require the Matlab Statistical Toolbox, but this component of the method is not essential for anything else. No additional toolboxes are needed for any of the fuzzy logic or evolutionary algorithm execution. Pseudorandom numbers are generated using the internal Matlab function "rand".

## Authors' contributions

S.D. implemented the method and performed simulations described in the manuscript. He was also was the primary author of the manuscript text. B.A.S. was responsible for originally developing the method described within, actively guided S.D. during his work, and made major contributions to the final text. Both authors read and approved the final manuscript.

## Supplementary Material

Additional file 1 **Supplementary Table 1**. Fuzzy rule sets obtained (using the TT3 data set) for each cell cycle gene, showing each run of the evoluationary algorithm (with a different random number generator seed). **Supplementary Table 2**. Rule sets obtained for each gene (a) using the TT3 data set (lowest error ("best") and consensus of algorithm runs, (b) using the TT2 data set, and (c) using the Shake data set (with only the consensus shown for the latter two). **Supplementary Table 3**. Errors and probabilities of finding rule sets by chance for each data set, applying the rule sets shown in Supplementary Table 2. **Data Sheets**. In addition, there are sheets with the data sets described in the paper, containing fuzzified, arctan(log)-transformed gene expression ratios for the human cell cycle marker genes (Table 2). Click here for file
